# 3D Printing Decellularized Extracellular Matrix to Design Biomimetic Scaffolds for Skeletal Muscle Tissue Engineering

**DOI:** 10.1155/2020/2689701

**Published:** 2020-11-17

**Authors:** Silvia Baiguera, Costantino Del Gaudio, Paolo Di Nardo, Vittorio Manzari, Felicia Carotenuto, Laura Teodori

**Affiliations:** ^1^Department of Fusion and Technologies for Nuclear Safety and Security, Diagnostic and Metrology (FSN-TECFIS-DIM), ENEA, Italy; ^2^Department of Clinical Science and Translational Medicine, University of Rome “Tor Vergata”, Italy; ^3^Interdepartmental Center for Regenerative Medicine (CIMER), University of Rome “Tor Vergata”, Italy; ^4^E. Amaldi Foundation, Via del Politecnico snc, 00133 Rome, Italy; ^5^L.L. Levshin Institute of Cluster Oncology, I. M. Sechenov First Medical University, 119991 Moscow, Russia

## Abstract

Functional engineered muscles are still a critical clinical issue to be addressed, although different strategies have been considered so far for the treatment of severe muscular injuries. Indeed, the regenerative capacity of skeletal muscle (SM) results inadequate for large-scale defects, and currently, SM reconstruction remains a complex and unsolved task. For this aim, tissue engineered muscles should provide a proper biomimetic extracellular matrix (ECM) alternative, characterized by an aligned/microtopographical structure and a myogenic microenvironment, in order to promote muscle regeneration. As a consequence, both materials and fabrication techniques play a key role to plan an effective therapeutic approach. Tissue-specific decellularized ECM (dECM) seems to be one of the most promising material to support muscle regeneration and repair. 3D printing technologies, on the other side, enable the fabrication of scaffolds with a fine and detailed microarchitecture and patient-specific implants with high structural complexity. To identify innovative biomimetic solutions to develop engineered muscular constructs for the treatment of SM loss, the more recent (last 5 years) reports focused on SM dECM-based scaffolds and 3D printing technologies for SM regeneration are herein reviewed. Possible design inputs for 3D printed SM dECM-based scaffolds for muscular regeneration are also suggested.

## 1. Introduction

The reconstruction of skeletal muscle (SM) due to volumetric muscle loss (VML) still remains a complex and unsolved task, and the development of *ad hoc* strategies to promote functional tissue regeneration, following muscular traumas or disease, is a real need [1, 2].

To develop muscle grafts that actually reconstruct and restore SM large-scale injuries, different tissue engineered strategies have been developed; however, a suitable biomimetic solution to obtain functional muscular constructs has not, till now, be found. Therefore, novel approaches that can facilitate safe bigger muscle tissue repair and regeneration should be developed [3].

The ideal biomaterial should fill the VML, including the muscular basal lamina, sustain cells/stem cells activity, and promote cellular orientation, alignment, and maturation, allowing access to vascular and neural cells [4, 5]. For this aim, dealing with the tissue-specific extracellular matrix (ECM), properly treated, can represent a suitable option to define ad hoc therapeutic protocols. The ECM, indeed, contains numerous bioactive molecules, such as growth factors and cytokines, regulates cellular activities, and provides a physical ultrastructure that accommodates peculiar cell types [6–8]. Moreover, it has been demonstrated that ECM basal lamina plays an essential role in the regeneration process, acting as a tissue template and secreting chemotactic factors for stem cell recruitment [9–11]. For these reasons, ECM-based scaffolds, acting as regenerative templates and modulating the healing process, seem to be one of the most promising and interesting means to support muscle regeneration and repair [12]. Moreover, using ECM-derived scaffolds to generate new organs for transplantation has been suggested as a potential method to be effectively considered, and it has been included among the 10 most significant developments in the last 30 years [13].

In this regard, also the selection of the fabrication technique is pivotal to positively drive the cell response guided by the scaffold architecture. Additive manufacturing (AM) has recently emerged as a valuable methodology to produce geometrically defined three-dimensional structures, significantly improving their physiological relevance through the architectural mimicking of native tissues and organs. Particularly, 3D printing technology overcomes major drawbacks of conventional scaffolding techniques, including the limited control over the 3D structures of engineered tissues and the reduced reproducibility [14]. In this context, stereolithography, for instance, can usefully support the fabrication of biomimetic scaffolds for muscle repair, being characterized by the highest resolution level and being thus capable to fabricate aligned structural elements with the characteristic size of muscle fibers.

With the aim to identify innovative biomimetic solutions to develop engineered muscular constructs for the treatment of SM loss, the more recent (last 5 years) approaches including SM dECM-based scaffolds and 3D printing technologies for SM regeneration are herein investigated. Moreover, possible design inputs for 3D printed SM dECM-based scaffolds for muscular regeneration are also suggested. This review was therefore structured to briefly introduce the skeletal muscle tissue and its specific characteristics. Subsequently, in order to address VML issues, the potential role of the SM dECM as an instructive naturally derived material and the related 3D printing methodologies capable to process it for fabricating a biomimetic scaffold are discussed.

## 2. Skeletal Muscle Tissue

With more than 600 different muscles, SM constitutes about 40% of the human body mass, and it is the most common muscle tissue. It consists of long, parallel, multinucleated cells (muscle fibers) wrapped by a thin connective tissue (endomysium) and bounded together by collagenous sheets (perimysium), forming the fiber bundle. Several muscle bundles, enveloped together by a thick collagenous external sheath (epimysium), form a muscle. Muscle fibers may range from 10 to 100 *μ*m in diameter and from a few millimeters to many centimeters in length [15]. They are highly specialized to produce force and movement and are connected both to the vascular network, for constant nutrients and metabolite delivery and waste removal, and to the neuronal network for activation and contraction. Functionally, muscle fibers are often distinguished between slow and fast fibers depending on their metabolic activity (respectively, oxidative, or glycolytic metabolic pathways) and on the myosin heavy chain proteins within the contractile fiber apparatus [16]. SM fibers appear striated due to the alignment of repeated functional contractile units called sarcomeres, which consists of interposed filaments of actin and myosin [17].

SM tissue is characterized by a regenerative capacity, due to the activation of progenitor muscle cells (satellite cells), located underneath the basal lamina, which can fuse with healthy muscle fibers to regenerate and repair the damaged fibers. However, since SM fibers cannot divide, this repair property is limited only to small-scale injuries. When muscle mass loss is greater than 20%, muscular regenerative capacity is inadequate and may lead to extensive and irreversible fibrosis, scarring, and loss of muscle function [18]. Traumas associated with VML result then related to lasting functional impairment and impacts patient's life quality by significantly reducing the movement ability. While mild/moderate traumas can be treated using different established approaches [19, 20], severe and extensive muscle injuries often require surgical management [2, 21]. Although transposition flaps represent the current clinical gold standard for VML treatment [22], they are far from being ideal because of the risk for donor site morbidity, reduced muscular function, graft failure, and high costs [1, 21, 23]. Xenografts and allografts may eliminate donor-site morbidity and decrease operating time; however, they result associated with the risk of severe immune response, transmission of infective diseases, and slower integration with the native tissue [24].

Several tissue engineering strategies have been explored using a variety of materials, ranging from synthetic to natural polymers, or their combination, to decellularized ECM, or cell-based approaches (Table 1) [3, 11, 18, 25]. All of these strategies have pros and cons, and even if many have shown promising outcomes in terms of SM regeneration, the proper cellular microenvironment to bioengineer the SM construct has not, till now, be found [11, 18, 26].

## 3. Decellularized ECM-Based Scaffolds for SM Regeneration

Natural ECM is an heterogeneous microenvironment made of proteoglycans, proteins, and signaling molecules, providing architectural rigidity and mechanical support, regulating turgor pressure, forming intracellular connections, and modulating the binding sites and activity of growth factors (acting also as a local factor reservoir). The ECM composition influences matrix stiffness and rigidity (affecting cell differentiation, migration, and proliferation), permeability (affecting nutrient diffusion to tissues and cell function), and cell-matrix interactions (affecting cell adhesion and proliferation) [27]. As a consequence, the ECM acts as a structural and signaling microenvironment for cells, influencing cell behavior in terms of differentiation, proliferation, survival, and migration. Several studies demonstrated that, despite the improvements made till now, accurately mimicking the ECM complex structure is still lacking, and the reproduction of a scaffold capable to simulate complex tissues, such as SM, remains a technically unsolved issue [25, 28].

ECM-based materials provide an effective means for capturing this complexity and can assist as inductive templates for constructive remodeling [28]. Decellularization has been widely used for the development of ECM-based scaffolds, which retain the architecture and complexity of the native tissues, including vasculature and ECM biofactors [27, 29]. Moreover, it has been suggested that dECM-based bioscaffolds generate low molecular weight matricryptic oligopeptides with the ability to recruit and influence endogenous progenitor cells, playing a role in the constructive and functional remodeling process, including vasculature and innervation formation [30, 31]. Current models of VML have demonstrated the ability of ECM to stimulate a degree of neomyogenesis, confirming the critical proof of concept to use dECM for muscle regeneration [32].

Different tissue sources, both nontissue-specific and skeletal muscle, have been used so far to produce acellular scaffolds for the treatment of muscle loss.

### 3.1. Nontissue-Specific dECM

Small intestine submucosa matrix (SIS) and urinary bladder matrix (UBM) have been commonly considered, both for animal and clinical studies, as nontissue-specific ECM scaffolds for the treatment of muscle loss [26, 27, 33]. These scaffolds provided constructive tissue remodeling, including the formation of site-appropriate SM tissue, and promoted perivascular stem cell mobilization and accumulation within the site of injury [31, 34, 35]. Despite these positive results, reduced fiber generation, significant fibrotic tissue formation, and insufficient functional recovery have also been reported [36, 37].

SIS and UBM scaffolds are produced from thin tissues and not only do not have any muscular specific components, such as laminin *α*1 and *α*2 [38, 39], but they do not possess specific properties found in SM such as alignment and muscle-specific biochemistry as well [40]. Being ECM spatial arrangement, composition, and interaction with cells and growth factor tissue- and functional-specific, it is plausible to suggest that nontissue-specific ECM scaffolds can be unlikely suitable matrices for an appropriate muscular regeneration.

### 3.2. Skeletal Muscle dECM

3D architecture has a significant relevance for the regeneration of complex organs and tissues: in particular, the alignment of SM cells, allowing the formation of organized myotubes, is an essential topographic cue in musculoskeletal myogenesis [41, 42]. SM dECM retains its native morphology, supporting muscle healing and promoting a proregenerative immune response, implant integration, and tissue regeneration [43], and preserves the correct ECM architecture surrounding each myofiber [30]. On the basis of these considerations, SM ECM could play a critical role in acute regeneration, (i) orchestrating myoblast chemotaxis, proliferation, and fusion to form myotubes; (ii) releasing specific ECM growth factors and biomolecules that trigger satellite cell activation; and (iii) thus promoting myofiber differentiation, alignment and, ultimately, regeneration of functional SM [44].

**Figure 1:**
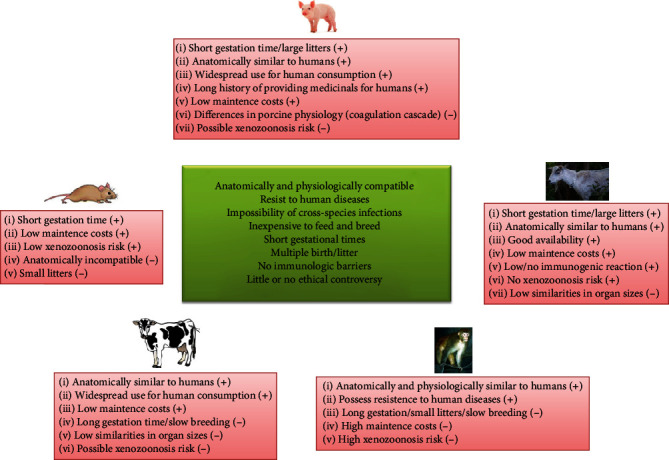
Animal properties to be organ donors for humans (central box) and pros (+) and cons (-) of different animal sources [45–48].

To date, different animal models have been used as tissue source to develop SM dECM. Each animal model has its pros and cons, and no one completely matches the needed properties to be organ donors for humans (Figure 1).

Most of the studies have focused their attention on porcine muscles, due to their anatomical and physiological similarities to humans. However, the risk of porcine endogenous retroviruses, which may integrate to the host genome, is inevitable [49]. Human-derived SM dECMs have been obtained with positive results [50, 51], even if the shortage of cadaveric donor organs significantly delays the obtainment of biological substitutes and increase patients' waiting time [52].

Different attempts to isolate and process SM dECM have been evaluated, and the main decellularization protocols, applied in the last years, are essentially of two types: detergent and detergent-enzymatic treatments (Table 2). Although most of these processes efficiently remove cellular components, at the same time, they can have negative effects on the composition, ultrastructure, biological activity, and biomechanical property of the remaining ECM, affecting the subsequent host response [73, 74]. Moreover, the effectiveness of the decellularization process depends also on animal source, muscle type, and dimensions.

To obtain a bioengineered muscular tissue construct, the resulting SM dECM has been used in a number of different ways: (i) as scaffold, maintaining the shape of the original tissue or organ; (ii) as hydrogel-type ECM; and (iii) as electrospun ECM-based structures.

Aulino et al. demonstrated that dECM scaffolds, guiding migration and differentiation of stem cells, could represent a suitable environment not only for myogenesis but also for cartilage and bone formation [75]. Orthotopic transplanted diaphragm-derived ECM supported a local immune response, activating a proregenerative environment and stimulating host muscle progenitor cell activation and migration [58]; moreover, the same decellularized scaffold was able to promote generation of new blood vessels, new muscle fibers, and most importantly, to partially recover host diaphragmatic function in a mouse model of congenital diaphragmatic hernia [61]. Promising results have been also collected dealing with abdominal dECM scaffolds aimed to treat partial thickness and full abdominal wall defects in a rat model [60, 70]. Furthermore, decellularized rat muscle matrix, characterized by an aligned structure, enhanced muscle function and regeneration in a large volumetric muscle defect, supporting the formation of new neuromuscular junctions and vascular networks [55, 56]. Decellularized human SM samples have been tested to close a surgical defect of the abdominal rectus muscle: muscle graft induced neovascularization together with initial proliferation of muscle fibers and migration of progenitor cells [76]. Even though neat SM dECMs have shown promise in VML models, there remain obstacles in modulating physicochemical properties and scaling such materials to clinically relevant shapes and sizes. Many scientists have sought to overcome these limitations by enzymatically digesting these materials and, taking advantage of their natural thermoresponsive properties, fabricate scaffolds to be used as injectable hydrogel for promoting SM regeneration [53, 67]. For instance, SM dECM hydrogel combined with hyaluronic acid was used as a substrate for muscle progenitor cells and proved to be an optimal culture microenvironment potentially due to its similarity to the in vivo environment, suggesting a possible use for cell-based therapy for SM dysfunction [77]. Ungerleider et al. demonstrated that SM ECM-based hydrogels supported functional outcomes through altering key pathways associated with inflammatory response, cell death and survival, metabolism, and vessel and muscle development [68]. A nanofibrous SM ECM hydrogel allowed an improved myoblast viability, engraftment, and ischemic limb perfusion *in vivo* [54]. Although successful outcomes have been obtained to control size, shape, and structural integrity/stability of dECM hydrogel-based constructs (such as crosslinking modifications), the digestion required for their formation often inactivates important ECM components, and there is a limited control over the internal architecture of the material [78]. Electrospinning, allowing the fabrication of micro- and nanofibers tuning the diameter, alignment, and scaffold porosity [79, 80], has been used to collect an aligned structure that resulted similar to the anisotropic arrangement of stretched SM myofibers and provided a topographic cue for morphogenesis [25]. Smoak et al. developed a novel, high-throughput procedure to fabricate electrospun dECM scaffolds with tunable physicochemical characteristics, while maintaining the structural matrix components required for SM regeneration [63]. An electrospun scaffold, composed of SM dECM aligned nanofibers and polycaprolactone (PCL), supported satellite cell growth, myogenic protein expression, and myokine production [57]. The same scaffold, implanted in a VML murine model, modulated macrophage-mediated inflammation and increased myofiber regeneration. However, improvements in muscle weights and force production were not observed [81]. Recently, a multiscale composite scaffold, made of aligned electrospun dECM nanofibers, led to suitably align muscle cells alongside a nanosized ECM basal lamina [65]. While successfully, electrospun dECM-based scaffolds, due to relatively thin structures and low mechanical stability, cannot recapitulate the physiological microenvironment to bioengineer a three-dimensional volumetric SM tissue construct. To overcome this limitation, recent developments in 3D printing technologies can support the fabrication of volumetric tissue-like structures with a complex geometry in a layer-by-layer fashion.

## 4. Additive Manufacturing for SM Regeneration

3D printing technologies may allow to produce patient-specific implants with structural complexity, capable to mimic tissue morphological and biochemical cues [82]. The main advantage of this fabrication approach is related to the control that can be exerted on the processing variables from the design stage to the final product. These features can contribute to realize the expected microarchitecture by stacking several layers, generally reproducing a regular pattern. A regular 3D printed pattern is often considered a suitable option to control the expected functionality and cell distribution [83], but a tissue engineered scaffold should be regarded as a temporary substitute of the natural ECM of the tissue to be healed, resembling the complex tissue hierarchy, and this implies an effective biomimetic approach [84–86]. Following this rationale, a few studies are aimed at introducing a random microarchitecture to mimic tissue-specific ECM, e.g., bone tissue [87, 88], by means of 3D printing.

The potential of this approach can be usefully implemented for SM tissue engineering [89–91]. Topographical cues have been considered one of the main requirements to guide myoblast/stem cell response and induce myogenic differentiation and maturation, and anisotropic environment promotes cell alignment, their fusion, and myogenesis [92]. To complete the rational design of a tissue engineered construct, not only the biological characteristics have to be considered but also the scaffold processing conditions that obviously concur to the desired positive outcome. In this regard, among all the available AM technologies, stereolithography may offer a valid alternative to fabricate ad hoc scaffolds characterized by a higher level of morphological details, possibly enhancing the SM tissue engineering expectations. This fabrication option can finely mimic specific structural features of the tissue to be healed, thus providing a more biomimetic environment.

Combining specific AM approaches and viable biological components can pave the way to the development of bioactive scaffolds. Such a strategy can support the preparation of actual tissue engineered constructs, being already three-dimensionally biologically conditioned with respect to those undergoing to a postprocessing stage in terms of functionalization and cell seeding, which may not affect the whole structure.

### 4.1. Bioprinting dECM Scaffolds

The main technologies used for 3D deposition and patterning of biological materials in the bioprinting sector are inkjet, microextrusion, and laser assisted printing; each of them being characterized by different features related to surface resolution, cell viability, and biological materials to be processed. Inkjet printers allow to release controlled volumes of liquid at predefined locations by means of a number of delivering modes, such as thermal or piezoelectric, and can provide high cell viability, i.e., ≥85% [14, 91]. Microextrusion printers deliver a controlled volume of a material as a continuous strand by means of pneumatic or mechanical (piston or screw) systems, the most common mechanisms. Due to shear stresses, cell viability is generally lower (range survival rate 40–86%) [14]. Laser-assisted bioprinting relies on a ribbon made from glass which is coated with a laser-energy-absorbing layer (e.g., gold or titanium) and loaded with the bioink. A laser pulse focused on the metal film produces a high-pressure bubble that propels cell-containing materials toward the substrate; cell viability is usually high (≥95%) [91]. However, a suitable result is strictly related to fast gelation kinetics and compatibility of working wavelengths to preserve the resolution and arrangement of cells and biomaterials in 3D printed scaffolds [93].

In this framework, irrespective from the bioprinting technique, the bioink is the main actor of the experimental setup, whose characteristics, commonly those of a hydrogel, should be finely tuned and preserved. In addition, it should have the same composition and function of the native ECM which varies from tissue to tissue, and most of hydrogels for bioprinting unlikely provide this complexity [74]. The possibility to tailor the formulation of the bioink in order to promote a biomimetic response, combining not only cells, but also the tissue-specific ECM to modulate the critical cellular processes is a strategic key-point for a properly manufactured engineered scaffold.

Currently, natural and synthetic polymers are employed as bioinks for bioprinting SM constructs [91, 94]. Naturally derived hydrogels, such as collagen, alginate, and gelatin, have been used to provide physical support and cell instructive functionalities; others, such as calcium alginate or fibrin, characterized by fast crosslinking properties, have been used directly as bioink or as a supporting polymers [91]. Natural hydrogels can promote cell growth, are tunable, and characterized by biodegradable properties; however, they lack the specific mechanical features necessary for a suitable muscle regeneration. Due to their good mechanical strength, synthetic polymers, such as PEG-based hydrogels, poly(lactic-co-glycolic acid), PCL, PVA, and polyurethane have been frequently considered for bioprinting SM constructs [91, 94]. The conjugation with functional groups resulted necessary to make synthetic polymers photocrosslinkable; while to enrich the scaffold with cell supportive properties, composite bioinks of natural–synthetic polymers have been used [91].

Most of the prepared hydrogels for bioprinting does not show the structural, chemical, biological, and mechanical complexity of natural ECM microenvironment for cells engraftment, survival, and function. Therefore, dECM, due to its unique tissue-specific composition and topology, and containing peculiar proteins, important signals for cell fate, could be the optimal material for preparing biomimetic bioinks [47, 73, 74].

Several studies investigated the topic, showing the potential of this approach to design novel and active biomaterials, assessing dECM from different tissues, e.g., porcine cartilage and heart tissues and human adipose tissue [95], kidney [96], bovine Achilles tendon [97], or porcine heart mechanically tailored by using vitamin B2 and UVA irradiation followed by thermal crosslinking to resemble native cardiac tissue and promote cardiac differentiation of progenitor cells [98]. Focusing on SM tissue engineering, Choi et al. prepared a bioink including porcine SM dECM and C2C12 myoblasts to be processed by means of in-house developed 3D cell-printing system [71]. PCL was deposited at both ends of the construct as a geometrical constraint to induce cell alignment, which was also dependent on the printed linewidth of the scaffold. Authors showed that the highest alignment was obtained for diameters of 500 *μ*m, and as previously reported, the possible implication of the shear force, generated at the nozzle during the printing process, was assessed by cell viability tests which resulted in a minimal cell death after 24 h. In addition, mechanical properties were improved as well when compared to the control group [71]. This study substantially reported the suitability of the SM dECM with no particular mention to possible drawbacks. Porcine SM dECM was further investigated as an active means for bioink preparation, considering a methacrylation process to obtain a photocrosslinkable bioink, to be processed by means of a three-axis printing system equipped with a 30G single nozzle [66]. The printable material also included fibrillated PVA, as a sacrificial polymer to fabricate a uniaxially oriented patterned structure, and C2C12 myoblasts that were responsive to this culturing strategy. PVA concentrations affected the cell response, since high cell viability (>94%) was assessed for a polymer content of 5 and 10 wt%, while a low cell viability (27.2 ± 4.1%) resulted for a PVA concentration of 15 wt%, due to the high viscosity of the bioink. A more comprehensive approach assessed the potential of dECM from porcine tibialis anterior muscle and descending aorta, including human SM cells and human umbilical vein endothelial cells, respectively, to be printed in a mixed solution or coaxially [72]. The constructs were fabricated via an in-house developed 3D cell printing system, named the integrated composite tissue/organ building system, at 18°C to prevent dECM bioink gelation. The experimental plan intended to treat VML issues (about 40%) in a rat model and showed that prevascularized muscle constructs, coaxially fabricated, successfully mimicked the hierarchical architecture of vascularized muscles. Improved de novo muscle fiber formation, vascularization, innervations, and 85% functional recovery in VML injuries were collected with respect to constructs consisting of only muscle cells and SM dECM bioink and constructs made by mixing human umbilical vein endothelial cells, muscle cells, SM dECM, and vascular dECM bioink, both used for comparison.

### 4.2. Stereolithography of dECM Scaffolds

Stereolithography is an AM technique which relies on the layer photopolymerization of specific material by means of light irradiation, usually UV, according to a CAD input. Commonly to all 3D printing methodologies, the final structure is the result of stacked layers as the build stage is vertically translated. This working principle can be implemented in two different modes: in the first one, the movement of the light source is computer controlled to precisely polymerize each layer of the structure, while in the other one, called digital micromirror device, an array of thousand micromirrors can polymerize a whole layer at once by controlling each of them to reflect light in a spatial pattern [90]. Stereolithography is characterized by the highest resolution of all the bioprinting methods (~6 *μ*m), which can be further improved by the two-photon polymerization-based stereolithography (~200 nm). Polymerization is achieved by focusing two consecutive photons within the focal volume of a laser beam, and therefore, the polymerization threshold is not reached out of the focal volume. Typically, this technical approach allows to deal with treated volume less than 1 *μ*m^3^ [99]. Two-photon polymerization promoted the fabrication of scaffolds with nanoscale features similar to those of natural ECM, and this enables the possibility to further evaluate the cell response to this kind of environment [100].

Respect to other 3D printing technologies, stereolithography has several advantages as the scaffold external geometry and internal architecture can be finely controlled due to the intrinsic high resolution, being related to the spot size of the light source, and complex scaffolds can be thus easily fabricated [82], also allowing to modify printed scaffolds to add further functionalities [101, 102]. However, stereolithography is affected by some drawbacks as well. Only photopolymerizable solutions containing UV-activated photo-initiators can be processed, and the potential cytoxicity is a crucial issue to be addressed with polymeric suspension including cells, still representing a possible limitation also for acellular scaffolds due to the possible presence of unreacted compounds. Moreover, cells are exposed to UV radiation, and this may impair their functionality and affect viability.

In terms of scaffold manufacturing, currently no 3D printing technology can be considered as the optimal option to realize a functional tissue or organ. Stereolithography has the highest resolution but lacks scalability and detailed investigations on photo-initiated cell damage, and the long-term effects of laser/UV radiation on cells represent a real need. Droplet-based systems can precisely pattern cells, but bioprinting human scale tissue is still an onerous task. Extrusion-based bioprinting has the least resolution of all the 3D printing technologies but has the highest potential to bioprint human scale tissues and organs [90].

To obtain biomimetic stereolithographic scaffolds, cell-laden photopolymerizable solutions, including not only cells but also specific biochemical compounds, like tissue-specific ECM, have been currently tested. In this regard, Chen et al. fabricated a pig cartilage dECM/gelatin methacrylate/exosome scaffold with radially oriented channels using desktop-stereolithography technology [103]. Scaffolds were then implanted into osteochondral defects in New Zealand white rabbits, finding that they contributed to restore cartilage mitochondrial dysfunction, enhance chondrocyte migration, and polarize the synovial macrophage response. Elomaa et al. proposed to replace gelatin methacrylate with methacryloyl-functionalized rat liver dECM, due to its difficult viscosity control [104]. However, this formulation was not assessed as the study focused on printability and subsequent characterization of 3D printed acellular human small intestine-mimicking tissue scaffolds made of gelatin methacrylate/poly (*ε*-caprolactone) methacrylate, and only a printed test case was shown. Biomimetic microarchitectures were proposed by Yu et al. to pattern cell-laden 3D dECM heart and liver tissue constructs for promoting the maturation of human-induced pluripotent stem cell- (hiPSC-) derived cardiomyocytes and hiPSC-hepatocytes [105]. The issue to deal with a photocrosslinkable dECM was addressed, showing that the fabricated scaffolds guided spontaneous cellular reorganization into predesigned striated heart and lobular liver structures through biophysical cues.

Further studies exploiting the potential of stereolithography should be carried out in order to prepare bioactive scaffolds accurately mimicking SM ECM properties as, to date and to Authors' best knowledge, no investigations included this biological structure in the preparation of the photocrosslinkable solution. The higher resolution allowed in the processing stage can effectively support the development of experimental protocols properly tailored to deal with instructive means for this specific tissue regeneration.

## 5. Future Perspective

**Figure 2:**
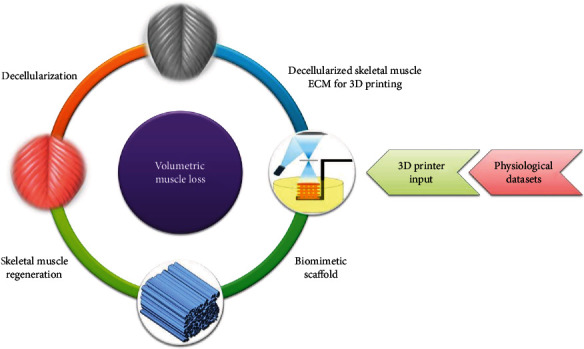
Schematic proposal of a 3D biomimetic scaffold for SM regeneration. Physiological datasets, e.g., histological images of SM fibers, may be the starting point to elaborate a CAD model reproducing the tissue configuration and then provide an ad hoc input to be processed by 3D printing, e.g., stereolithography, using SM dECM.

The interest toward AM for tissue engineering applications can actually contribute to open novel routes to address critical regenerative issues and provide alternative approaches to respond to urgent clinical needs. In this framework, biomimetics can play a pivotal role to support an effectively healing process, and the design of novel scaffolds should be thus accurately tailored. VML treatment can be planned defining ad hoc protocols based on the selection of tissue-specific biomaterials and 3D printing techniques capable to process bioactive tissue-derived materials. The analysis here presented may support an experimental study in which the dECM from SM tissue can be the suitable biomaterial for stereolithography manufacturing, aimed to reproduce the fine morphological characteristics of muscle fibers. Physiological datasets, for instance, may be the starting point to elaborate a CAD model reproducing the tissue configuration and representing an ad hoc input for 3D printing (Figure 2).

Tissue-specific dECM can provide the structural properties to match those of the natural tissue and all the necessary biochemical cues to design engineered scaffolds. For this aim, stereolithography seems to be one of the most promising manufacturing technique, thanks to its inherent properties even if an in-depth analysis is still necessary to verify the expected reliability.

## 6. Conclusions

AM can be a valuable option to design and fabricate biomimetic scaffolds with the aim to improve the expected outcome for tissue engineering applications. As here shown, focusing on SM regeneration, the tissue-specific dECM can be properly elaborated to be 3D printed as a structural and bioactive material for scaffold preparation. Cells, ECM, and biological compounds can be encapsulated into a supportive hydrogel to formulate a tailored bioink which allows to control the microstructure and three-dimensionally locate instructive inputs, being both two fundamental key-players of the tissue engineering paradigm.

The here presented paper reports the state-of-the-art for the SM regeneration, showing at the same time possible issues to be addressed and critically discuss the potential of AM for clinical needs. Bioprinting and stereolithography can provide significant evidences to develop promising therapeutic strategies, thanks to a more direct implementation of the first technique and to an intrinsic process resolution of the latter one that may contribute to effectively mimic the tissue-specific ECM.

## Figures and Tables

**Table 1:** Current strategies for SM regeneration.

Strategies	Materials	Engineered approaches	Pros	Cons
Natural scaffold-based	Alginate Chitosan Collagen Fibrin Hyaluronic acid Laminin	Fibrous meshes Hydrogels Porous scaffolds Sponges	Biocompatible Intrinsic bioactive signaling cues Facilitated controlled release of growth factors Can be configured in different forms Can be chemically modified	*In vivo* rapid degradation Do not generate uniform cell alignment and supported disorganized repair of large muscle defects Limited mechanical stiffness
Synthetic scaffold-based	Poly (glycolic acid) Poly (lactic acid) Poly-*ε*-caprolactone Poly(lactic-co-glycolic acid) Polydimethylsiloxane Polyurethane Copolymers (e.g., PLLA/PLGA)	Fibers Fibrous meshes Micro-/nanopattered substrates Microspheres Porous sponge-like scaffolds	Possess precisely tuned mechanical and structural properties Flexible in chemical and physical modification Reproducibility in preparation, modification, and chemical properties Readily fabricated into a variety of geometries Availability of various processing technologies allowing the fabrication of tissue shape and size-specific scaffolds with control on mechanical, structural, and physicochemical properties	Low bioactivity Need functionalization to improve cell attachment or regenerative outcomes Possible foreign body response
Decellularized scaffolds	Small intestine submucosa Urinary bladder Muscle-derived	As it is Hydrogels Minced tissue (for muscle)	Retain ECM architecture and complexity, including vasculature and biofactors Angiogenic, promotes vascularization Significantly improve functional outcomes	Decellularization process can significantly damage ECM structure and protein/growth factor content Incomplete decellularization can induce an inflammatory response
Cell-based	Mesenchymal stem cells Mesoangioblasts Myoblasts Pericytes Satellite cells	Systemic injections Seeded/loaded/injected on scaffolds/hydrogels Encapsulated in microspheres	Promote muscle regenerative capability Can form new muscle fibers	Low cell viability Poor cell migration and engraftment Need of immunosuppressive therapy Inefficient methods of delivery High costs for cell expansion and manipulation
Molecular signaling based	FGF HGF IGF-1 PEDF SDF-1a TGF-*β*1 VEGF Antisense specific nucleotides (e.g., nusinersen)	Systemic injections Incorporated in scaffolds/hydrogels	Activate and/or recruit host stem cells Enhance myogenesis Promote angiogenesis Functional recovery with revascularization	Short factor half-life Difficulty in controlled release

**Table 2:** Decellularization protocols for skeletal muscle dECM obtainment reported in the last 5 years.

Method	Materials	Muscle	Results	Ref
Detergent	Sodium dodecyl sulfate Triton X-100 Sodium deoxycholate	Porcine skeletal	Suitable decellularization using only SDS Production of a too thin gel for hydrogel	[53]
		Porcine major psoas	Suitable decellularization	[54]
		Rat gastrocnemius	Unaltered ECM anisotropy and chemical components	[55]
		Rat hind limb	Suitable decellularization Loss of specific sarcolemma proteins	[56]
		Bovine tail	Suitable decellularization	[57]
		Human flexor digitorum superficialis	ECM decellularized with unaltered composition	[51]

Detergent/enzymatic	Det + deoxyribonuclease Det + ribonuclease A Det + Trypsin	Rat, rabbit, human skeletal	Triton+trypsin resulted more effective in removing cellular material and maintaining the 3D fiber networks	[50]
		Rat diaphragm	Suitable decellularization ECM conservation, tissue micro- and ultra-architecture preservation	[58]
		Porcine skeletal	Suitable decellularization and gelation using trypsin/EDTA, Triton X-100, and Triton X-100/SDS	[53]
		Human rectus femoris and supraspinatus	Suitable decellularization	[59]
		Porcine rectus abdominal	ECM decellularized Altered ECM proteins levels	[60]
		Rat diaphragm	Suitable decellularization	[61, 62]
		Rabbit hind leg	ECM decellularized with unaltered collagen, proteins, and sGAG levels Loss of stiffness	[63]
		Rabbit lower limb	Suitable decellularization Altered collagen levels Increased pore sizes	[64]
		Porcine skeletal	Suitable decellularization Altered collagen, GAGs, and elastin levels No proper structural stability	[65]
		Porcine lower limb	ECM decellularized with altered collagen, elastin, and sGAG levels	[66]
		Human flexor digitorum superficialis	ECM not completely decellularized	[51]

Detergent/alcohol	Det + isopropanol Det + methanol	Porcine psoas	Suitable decellularization	[67, 68]
		Porcine longissimus dorsi	Suitable decellularization Difference in fat and protein compositions depending on harvesting conditions	[69]

Detergent/enzymatic/Alcohol		Porcine rectus abdominis	ECM decellularized Altered ECM proteins levels	[70]
		Porcine tibialis anterior	Suitable decellularization	[71, 72]

No detergent/no enzymatic	Latrunculin B Potassium chloride Potassium iodide	Rat hind limb	Suitable decellularization Loss of VEGF	[56]
		Human flexor digitorum superficialis	ECM not completely decellularized	[51]
